# Supplementary material for: The Phase Structural Evolution and Gas Separation Performances of Cellulose Acetate/Polyimide Composite Membrane from Polymer to Carbon Stage
Source: Membranes (Basel). 2021 Aug 12;11(8):618. doi: 10.3390/membranes11080618 (PMC8399511; doi:10.3390/membranes11080618)
Supplement: Supplementary file 1 [file membranes-11-00618-s001.zip › membranes-1319234-supplementary.pdf]

Supporting Information

The phase structural evolution and gas separation performances of cellulose acetate/polyimide composite membrane from polymer to carbon stage

Haojie Li[1,2], Shan Xu[2,3], Bingyu Zhao[1,2], Yuxiu Yu[1], Yaodong Liu[1,4*]

[1] Key laboratory of Carbon Materials, Institute of Coal Chemistry, Chinese Academy of Sciences, 27 Taoyuan South Road, Taiyuan 030001, China
[2] University of Chinese Academy of Sciences, 19 Yuquan Road, Beijing 100049, China
[3] State Key Laboratory of Coal Conversion, Institute of Coal Chemistry, Chinese Academy of Sciences, 27 Taoyuan South Road, Taiyuan 030001, China
[4] Center of Materials Science and Optoelectronics Engineering, University of Chinese Academy of Sciences, Beijing 100049, China
* Correspondence: liuyd@sxicc.ac.cn

Synthesis of PI

6FDA-DAM:DABA (3:2) polyimide (PI) was synthesized by two-step condensation including the preparation and chemical imine formation of polyamic acid (PAA). First, DAM (4.508 g, 0.03 mol) and DABA (3.043 g, 0.02 mol) were dissolved with NMP (119 ml) with a persistent stirring in nitrogen atmosphere. After complete dissolution, 6FDA (22.212 g, 0.05 mol) was slowly added into the mixed solution at - 10 °C and reacted at room temperature for 24 hours to prepare polyamic acid. Then acetic anhydride (47.25 ml, 0.5 mol) and 3-methylpyridine (4.85 ml, 0.05 mol) were successively injected into the above reaction mixture, and then reacted for 24 h to complete the imidization of PAA. 6FDA-DAM:DABA (3:2) polyimide solution was successfully synthesized. Finally, PI was prepared by precipitation of polyimide solution in methanol. The obtained polymer was dried in vacuum at 100 °C for 24 hours. The Figure S1 shows the 1H NMR spectrum of PI. The relative peaks corresponding to the proton is clearly shown, and each peak area is integrated to estimate the mole ratio of DAM to DABA. The calculated result shows that there is rational successful synthesis of PI.

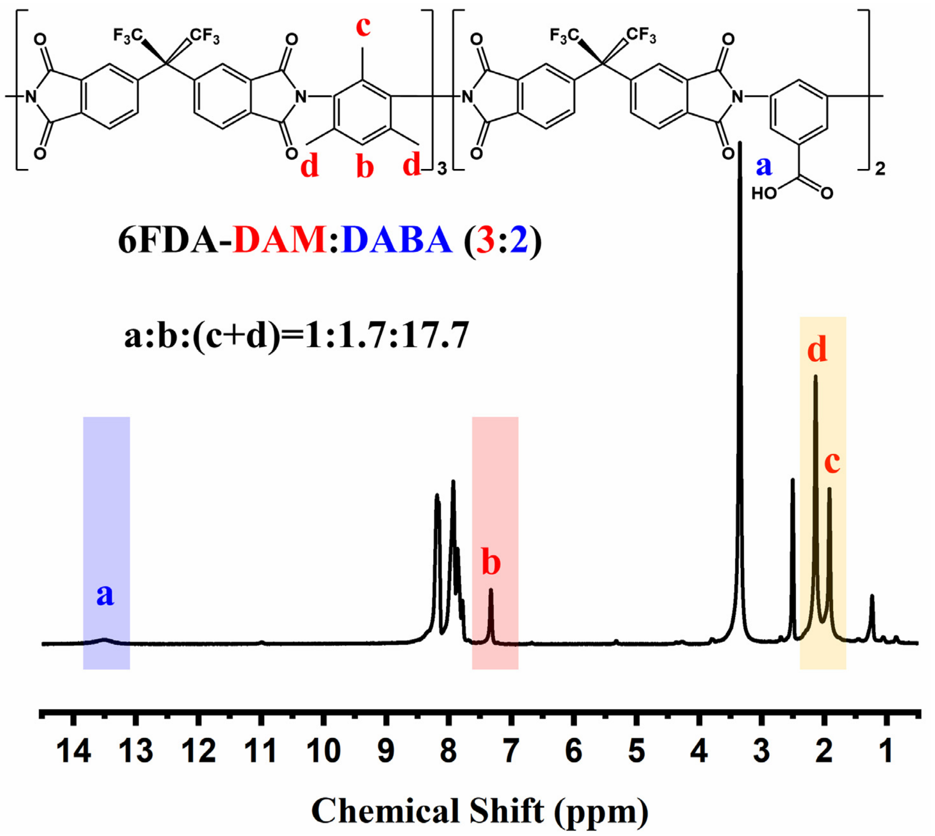

Figure S1. ^{1}H NMR spectrum of PI.

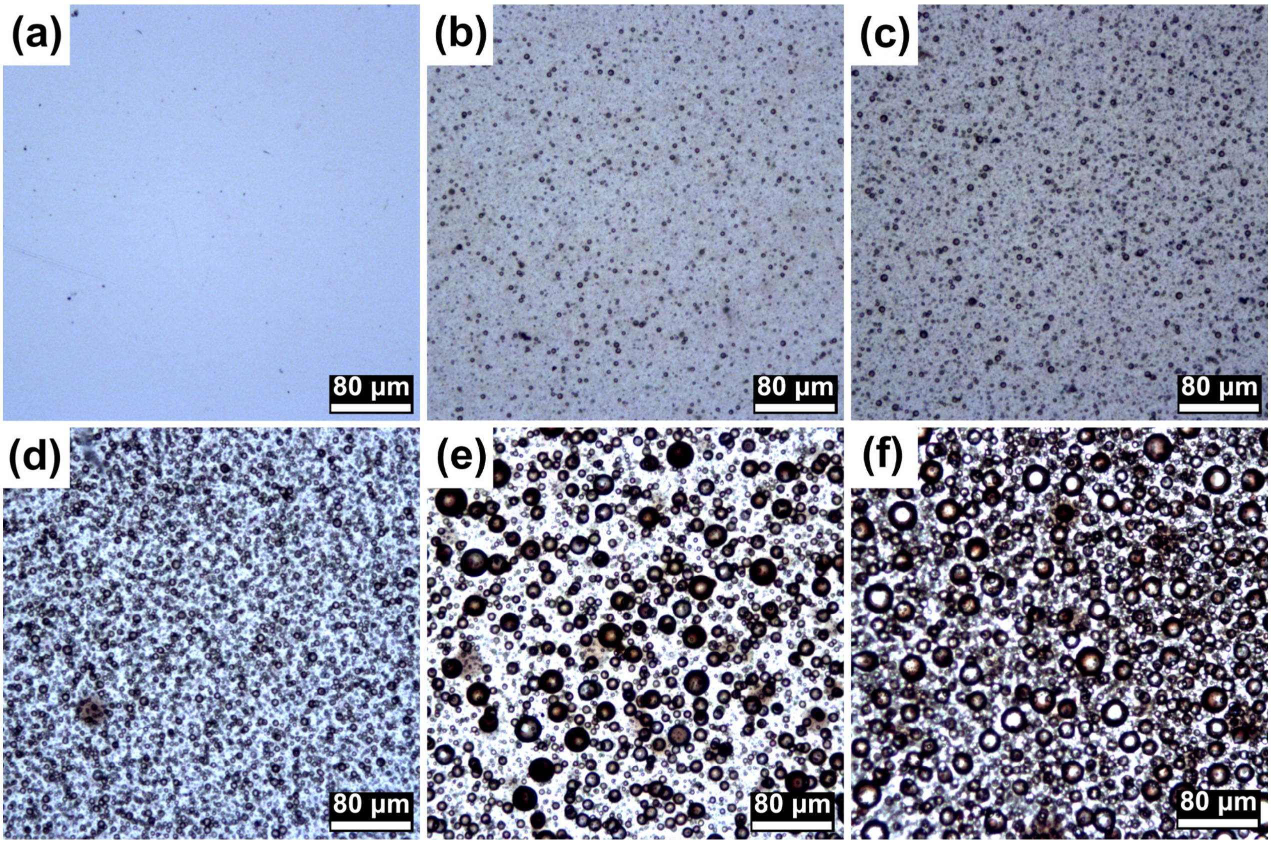

Figure S2. Optical images of membranes: a, b, c, d, e, f membranes of PI, CA0.5, CA1, CA5, CA10, CA20 at 350 °C, respectively.

Table S1. T_g of composite membranes.

**Sample**	**T_g (°C)**				
	**0.1**	**0.5**	**1**	**5**	**10 (Hz)**
PI	391.31	397.93	399.43	410.84	415.33
CA0.5	392.82	404.90	408.46	413.24	415.63
CA1	392.86	402.44	408.13	410.83	413.25
CA5	395.99	405.01	409.83	418.25	420.63
CA10	404.08	410.68	413.98	426.30	427.20
CA20	397.50	408.61	412.21	420.33	423.37

Table S2. The pore structure parameters of composite membranes from N_2 sorption.

Sample	S_{BET} (m^2g^{-1})	S_{mic} (m^2g^{-1})	V_t (cm^3g^{-1})	V_{mic} (cm^3g^{-1})
PI	178.05	<1	0.15	<0.01
CA1	68.87	<1	0.06	<0.01

Table S3. The pore structure parameters of CMSMs from N_2 sorption.

Sample	S_{BET} (m^2g^{-1})	S_{mic} (m^2g^{-1})	V_t (cm^3g^{-1})	V_{mic} (cm^3g^{-1})
CA-600	507.99	355.35	0.29	0.18
PI-600	743.91	666.52	0.40	0.33

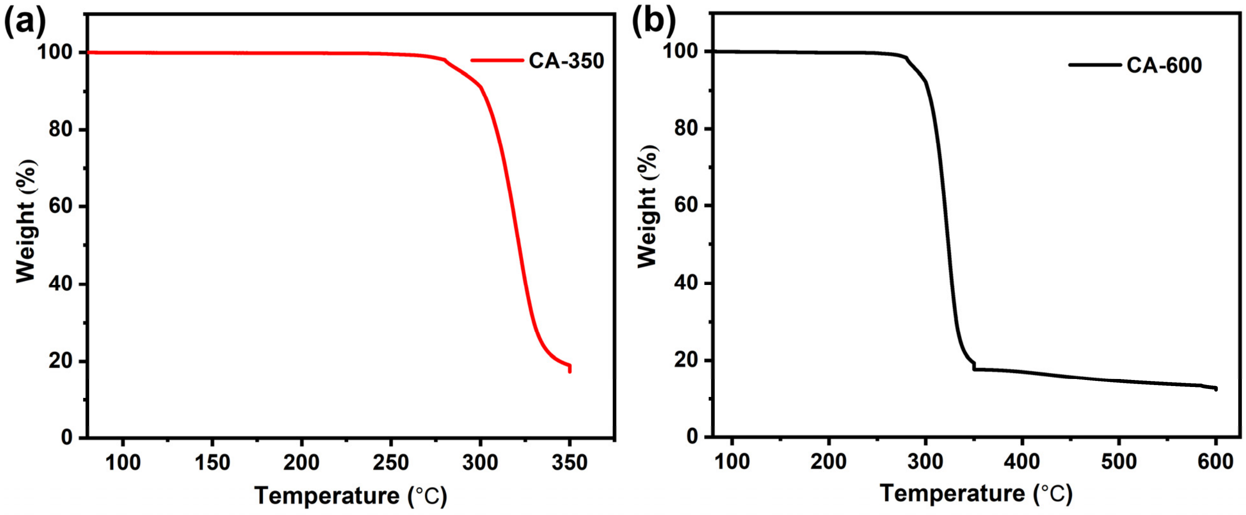

Figure S3. TGA curves of CA: (a) 350 °C; (b) 600 °C. The TGA procedure is the same as the pyrolysis procedure of the composite membranes.

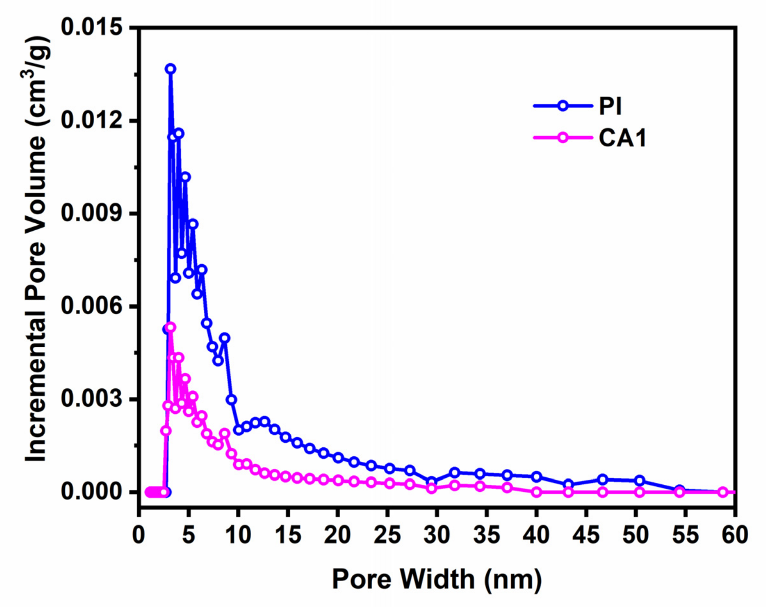

Figure S4. Pore size distribution curves: PI and CA1 based on N_2 sorption.

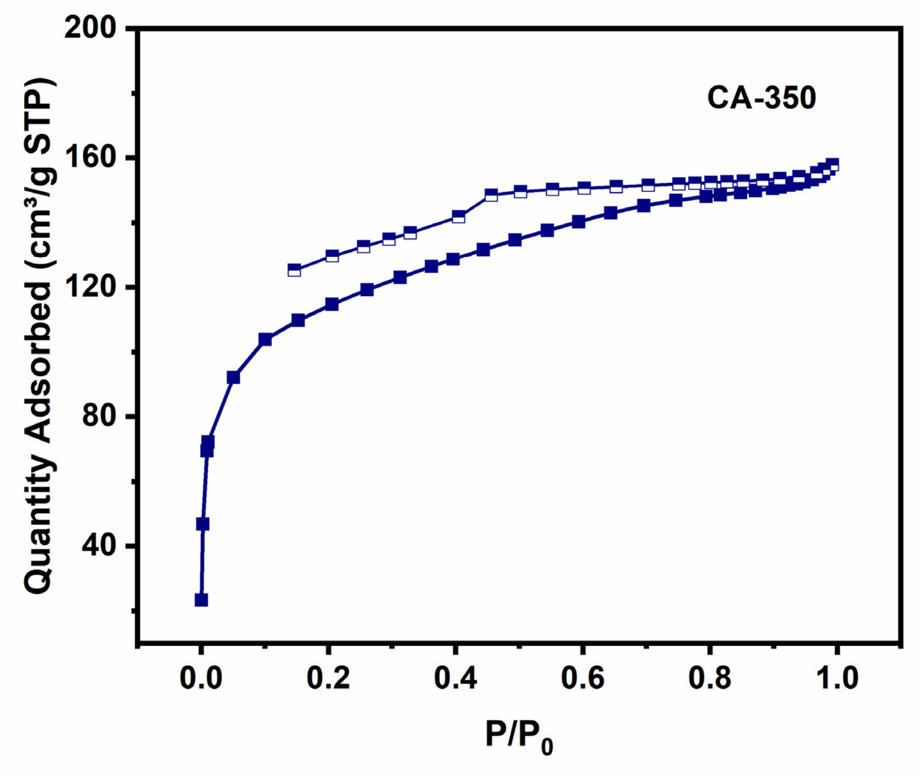

Figure S5. N_2 sorption isotherms curves of CA-350.

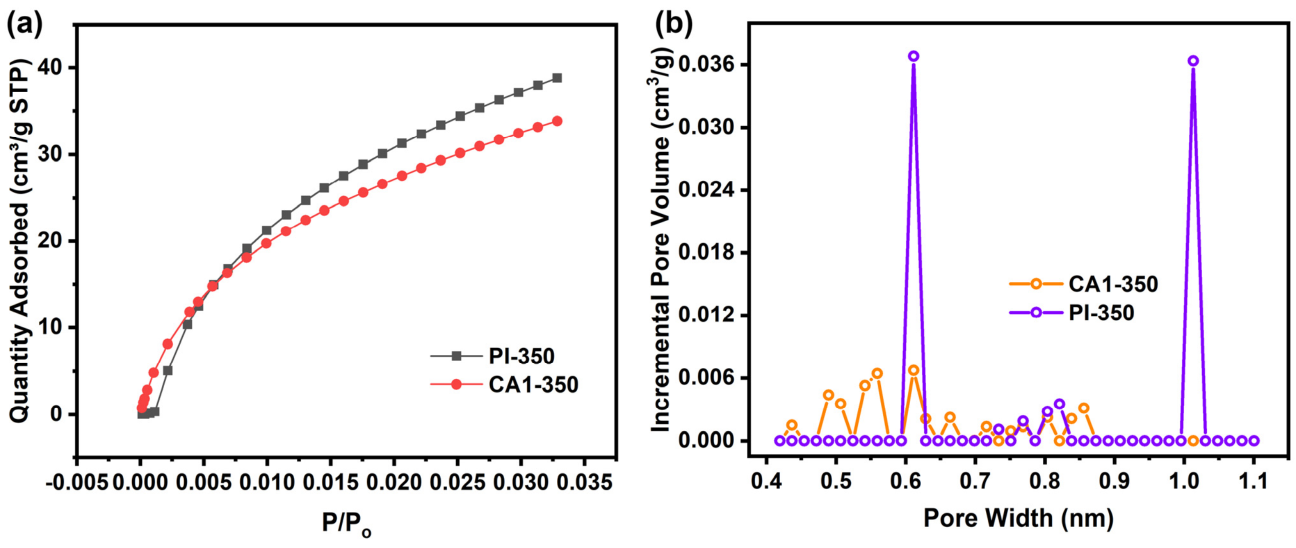

Figure S6. (a) CO_2 sorption isotherms curves of PI-350 and CA1-350; (b) Pore size distribution curves of PI-350 and CA1-350 with the 265 and 221 m^2/g BET surface area based on CO_2 sorption, respectively.

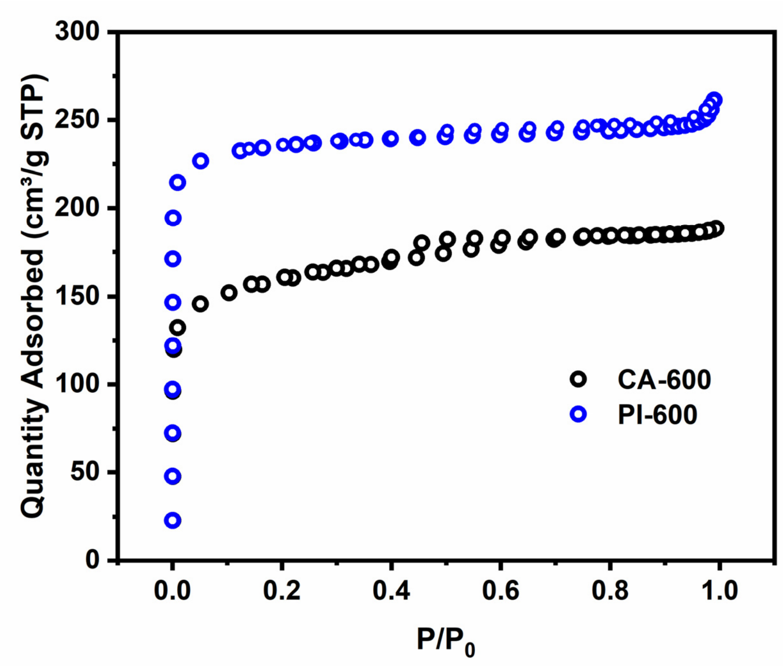

Figure S7. N_2 sorption isotherms curves of CA-600 and PI-600.

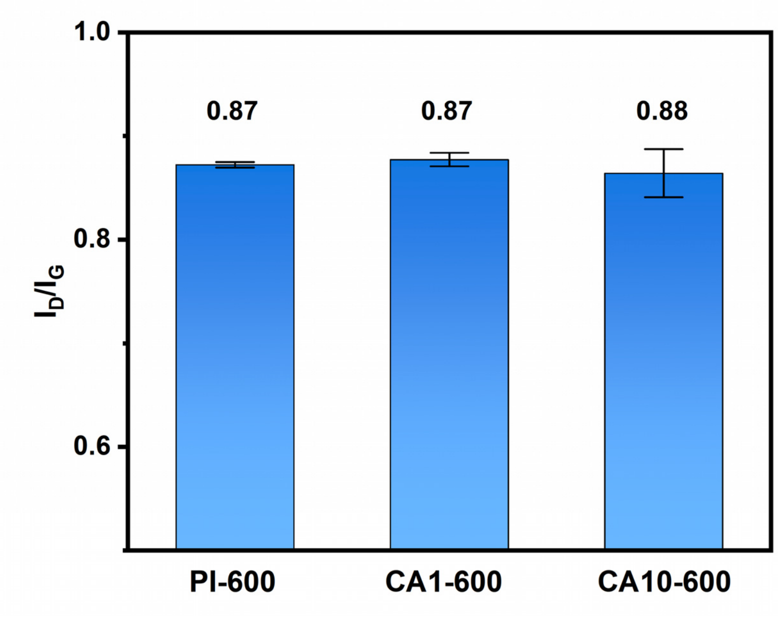

Figure S8. I_D/I_G of PI-600, CA1-600 and CA10-600 membranes.

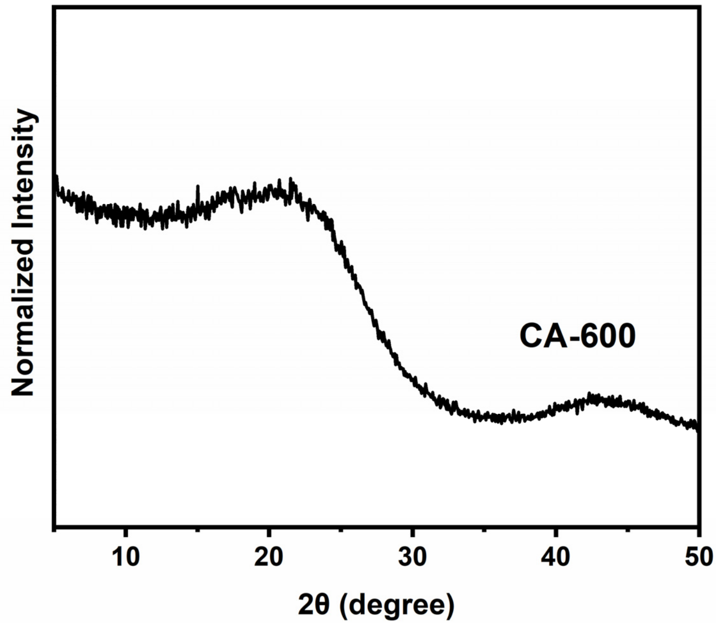

Figure S9. The wide-angle XRD patterns of CA-600.

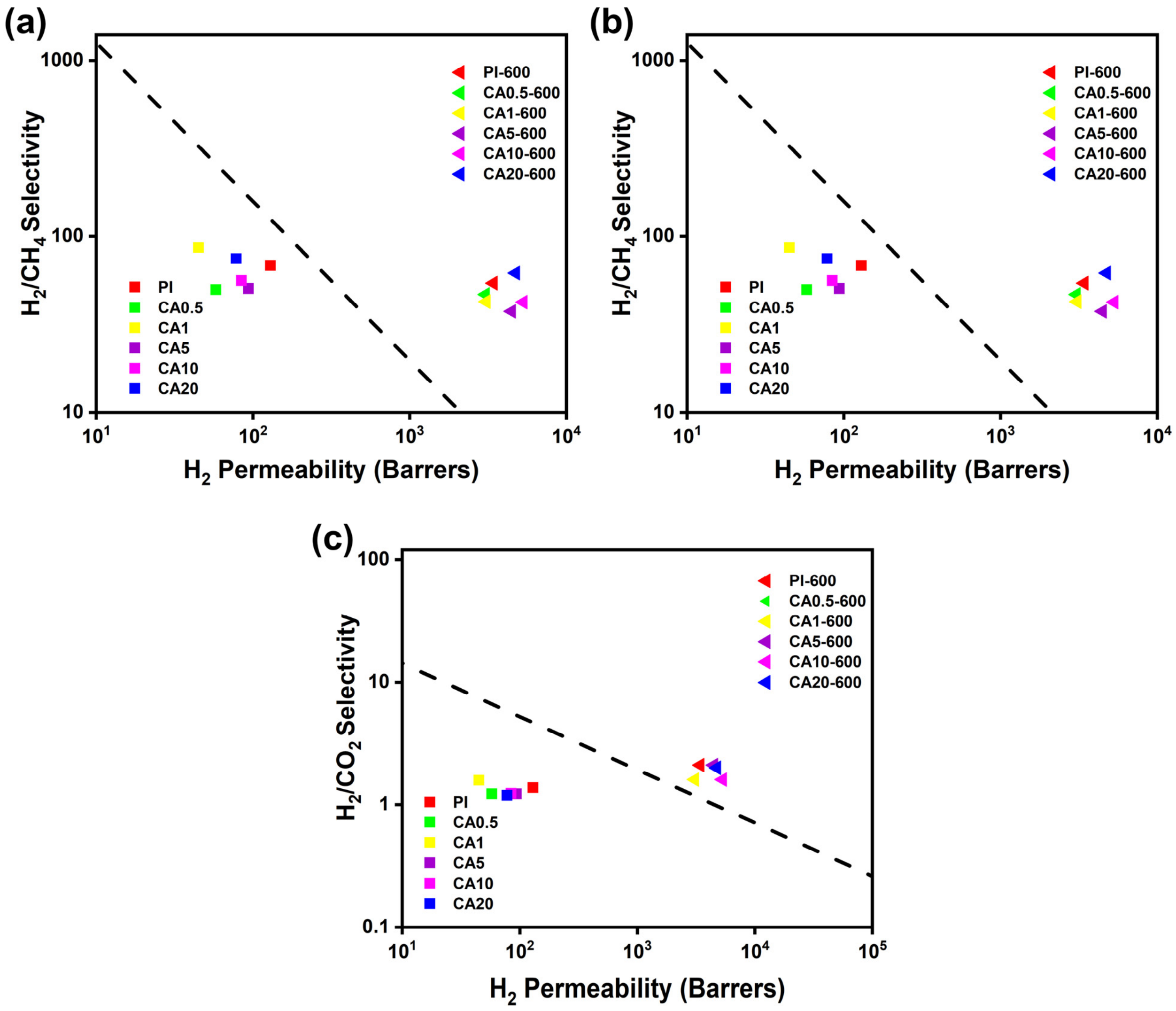

Figure S10. Separation performance for H_2 of the membranes prepared in this work with comparison to Robeson's upper bound presented in 2008.
